# Eucalyptus Disinfectant

**Published:** 1891-10

**Authors:** J. Brendon Curgenven


### EUCALYPTUS DISINFECTANT.

It was only a few years ago that the value of the eucalyptus tree as a preventive of ague and fever was made known. Since then it has been transplanted in districts addicted to this disease in this country, with beneficial results. Later on we are even assured that cures are effected by an extract of its leaves. Now we learn that the same agent is a powerful disinfectant and preventive of more fatal diseases. Dr. J. B. Curgenven writing to the *Farm and Home* (English), says of it: For the last two years I have used the "Eucalyptus Disinfectant" (a solution of anti-septics in the essential oil) in all cases of scarlet fever and diphtheria, and have not had one case of infection. In the former disease I have not used any isolation, and in most cases have not excluded the other children of the family from the sick room. None of the cases, except two or three severe ones, were kept to their room more than ten days, the isolation of six or eight weeks being unnecessary, as the cuticle is perfectly disinfected. This is accomplished by rubbing the disinfectant over the whole body twice, and then once a day for ten days. Baron von Müller, in a letter I received from him, quite approves of my method of disinfection by inunction.

It may interest your readers to know that I have used the disinfectant successfully in a case of "roup" in a hen by applying it to the mouth and throat by means of a camel's hair brush. From what I saw of the false membrane, covering the throat, cheeks and back of tongue, I believe roup to be diphtheria and as one of the sources of diphtheria in children. I mentioned this to a medical friend, and he recalled to mind a case of diphtheria he had attended in a family where they had several pigeons die of some throat affection. I am using it now on two hens that have lost the feathers on the back, breast and belly from some disease, probably of a fungoid nature, affecting the roots of the feathers. It has arrested the disease and young feathers are beginning to appear. I believe on further trial that it will be found very useful in all infectious diseases amongst domestic animals, as I have proved it to be in scarlet fever and diphtheria. All parasites on dogs, fowls, &c., are killed by it.

I am, sir, yours faithfully, J. BRENDON CURGENVEN.

